# Eye Masks and Earplugs to Improve Night Sleep Duration in Nulliparas: A Randomized Trial

**DOI:** 10.7759/cureus.32226

**Published:** 2022-12-05

**Authors:** Ik Hui Teo, Jesrine Hong, Peng Chiong Tan, Boon Kiong Lim

**Affiliations:** 1 Obstetrics and Gynecology, University Malaya Medical Centre, Kuala Lumpur, MYS; 2 Obstetrics and Gynecology, Faculty of Medicine, Universiti Malaya, Kuala Lumpur, MYS

**Keywords:** actigraphy, nullipara, pregnancy, sleep, earplug, eye-mask

## Abstract

Background

Women experience significant sleep disruption throughout pregnancy. Lack of sleep during the last month of pregnancy is associated with longer labor, a higher rate of Cesarean births, gestational diabetes, hypertension, and preterm birth. Eye-mask and earplugs through sensory deprivation increase sleep duration and quality in patients in the intensive care environment but their impact at home or during pregnancy is not known. We sought to evaluate eye-mask and earplugs compared to sham/placebo headbands on night sleep duration in pregnancy.

Materials and methods

A randomized trial was performed in a university hospital in Malaysia. Nulliparas between 34 and 36 weeks of gestation with self-reported night sleep of fewer than six hours were randomized to the use of eye-mask and earplugs or “sham” headbands during night sleep (both introduced as sleep aids). Night sleep duration was measured through a wrist actigraphy monitor during non-intervention week one and intervention week two with the allocated sleep aid.

Results

Data from 56 participants were analyzed on an intention-to-treat basis. Mean night sleep duration increased in intervention week two compared to non-intervention week one in both trial arms, which were 279 ± 19 vs. 304 ± 19 minutes (mean increase of 25 minutes) p = <0.001 and 286 ± 21 vs. 302 ± 22 minutes (mean increase of 16 minutes) p = <0.001 for eye-masks-earplugs and headband respectively. However, the mean increase in night sleep duration across trial arms (p=0.13) was not significant. A higher proportion of participants in the eye-masks and earplugs arm had their night sleep duration increased by at least 30 minutes, 13/29 (45%) vs. 5/26 (19%), relative risk (RR) 2.3 (95% CI 1.0-5.6) p = 0.04, more likely to agree that they slept better 19/29 (66%) vs. 7/27 (26%), RR 2.2 (95% CI 1.1-4.6) p = 0.03, expressed higher satisfaction score with their sleep aid 7 (7.0-7.5) vs. 6 (5-7), p = 0.003 and had lower induction of labor rates 4/29 (14%) vs. 12/27 (44%), RR 0.3 (95%CI 0.1-0.8) p = 0.02.

Conclusion

Eye masks and earplugs use in nulliparas with short night sleep duration in late pregnancy, lengthen their night sleep duration over baseline. Sleep is reportedly better and maternal satisfaction is higher with eye masks and earplugs use.

## Introduction

Women experience significant sleep disturbance and high rates of sleep disorder throughout pregnancy [1,2], especially in the third trimester [3]. Lack of sleep-in late pregnancy has a detrimental effect on pregnancy outcomes [4]. Nulliparous women who reported less than six hours of sleep per night during the last month of their pregnancy had a significantly longer mean duration of labor and were 4.5 times more likely to have cesarean births [5]. Women who slept less than seven hours at night are at increased risk of developing gestational diabetes, gestational hypertension, and preterm birth [6-8]. In women who had labor induction, a longer induction to delivery interval and higher cesarean delivery rate is associated with short night sleep duration in the last month of their pregnancy [9].

A 2004 report on sleep in late pregnancy, recommended that healthcare providers should assess sleep quantity, and sleep quality and prescribe at least eight hours of night sleep during pregnancy as these are potential predictors of labor duration and mode of delivery [5]. A 2015 study on sleep patterns and disturbances across pregnancy, suggested that all women should be screened and treated for sleep disturbances throughout pregnancy given the impact of inadequate sleep on fetal, pregnancy and postpartum outcomes are significant [1].

Noise and light, including those from the natural environment, are the two main causes of sleep disturbances even when not they are consciously observed, leading to sleep fragmentation and overall poor sleep quality [10]. Sensory deprivation with eye mask and earplugs (EMEP) increase sleep duration by 40%-60% and improve sleep quality and sleep architecture among patients in intensive care unit [11-14]. When used during the first night after surgery in a post-anesthesia care unit, EMEP also improved sleep quality, reduced the number of awakenings, and significantly decreased self-administered opioid use [13]. A systematic review on non-pharmacological interventions to improve sleep quality in pregnancy mentioned exercise, massage, and acupuncture (but not EMEP) did not yield definitive results [15].

A 2018 systematic review (19 studies involving 1379 participants) on the effect of EMEP in intensive care unit patients stated measuring the sleep quality or quantity is a major concern limiting objective comparison of results since non-standardized tools were used [11]. Polysomnography is considered the “gold standard,” for a sleep study, however, they are expensive, often requiring participants to sleep in a laboratory, hence limiting their usability [16]. Wrist actigraphy is an acceptable alternative objective method to assess sleep duration [17]. The wrist monitor delivers objective and accurate sleep or wake activity data over the course of days or weeks.

We hypothesized that EMEP use at home would improve actigraphy-derived night sleep duration among nulliparas in their late third trimester with short (less than six hours) [9] night sleep.

## Materials and methods

Trial design

This randomized controlled trial was approved by the Medical Ethics Committee of University Malaya Medical Center (date of approval December 14, 2017; Reference number 2017105-5648) and registered in the ISRCTN registry on December 28, 2017 (registration number ISRCTN28216612: https://doi.org/10.1186/ISRCTN28216612). The trial was conducted at University Malaya Medical Center with the first participant recruited on January 28, 2018 and the last on July 15, 2018. The trial was performed in accordance with the Declaration of Helsinki.

Participants

Nulliparas with no prior pregnancy beyond 20 weeks of gestation, aged ≥ 18 years, and singleton pregnancy, between 34 to 36 weeks' gestation with self-reported night sleep of fewer than six hours were identified for recruitment in the antenatal clinic during their regular visits. Exclusion criteria were pre-existing sleep disorder (e.g., chronic insomnia and sleep apnea), psychiatric disorder (e.g., depression, bipolar mood disorder, and schizophrenia), an underlying medical disorder that could affect sleep (e.g., epilepsy, heart disease, thyroid disorder, and lupus), night shift workers, active smokers, current alcohol consumption, obesity (Class II and above with body mass index > 35kg/m^2^), caretakers of dependent family members, intrauterine death or fetus with the gross anomaly. Our study recruited only nulliparous women to minimize potential bias from having to care for young children hence impacting sleep and out of concern that these dependents' call for attention at night might be hampered by EMEP.

Randomization and interventions

Eligible women were approached, provided with the patient information sheet, and verbally counseled regarding trial participation by the investigator. Informed consent was obtained from all participants. Participants were told that their self-reported short sleep would first be verified by a wristband actigraphy monitor (ActiGraph ActiSleep monitor, Pensacola, FL, USA) [18,19] over one week after which they would be randomized to a sleep aid, either EMEP or an elastic headband (HB) for the second week with the monitor.

We considered the elastic HB a sham or placebo intervention, but this was concealed from participants, and we characterized HB as a sleep aid (the ethics board consented to this approach) in order to evaluate the placebo impact in pregnant women of a suggested “sleep aid.”

Participants were provided with the wristwatch-like actigraphy monitor and instructed on its use. The ActiSleep monitor measures and records the frequency of human movement using a highly sensitive three-axis accelerometer and ActiGraph’s proprietary digital filtering algorithms. When compared to polysomnography, the wrist actigraphy monitor demonstrates a correlation (r) of over 0.8 for sleep duration, including studies of pregnant women [20,21].

Participants were asked to wear the device when they sleep at night for seven consecutive nights. The device must simply fit snuggly against the wrist and not be allowed to wobble around. Time into bed and time getting out of bed for sleep were recorded during the study nights as these data were required by the ActiLife (Pensacola, FL, United States of America) [18,19] software suite to calculate sleep duration.

Participants were asked to return after the non-intervention (baseline) the first week for sleep duration data download and they are only qualified to proceed into the trial for randomization if their mean night sleep duration were less than six hours and they were compliant with the study protocol i.e., provided a minimum of three nights’ sleep data [22] retrievable over the seven nights of recordings.

Randomization to EMEP or HB was carried out through the strict sequential opening of the lowest numbered available sealed and opaque envelope. Randomization sequences were prepared using a random number sequencer (random.org) in random blocks of four or eight and within the block by a co-author who was not involved in the recruitment process.

Participants randomized to EMEP were instructed to use them when they go to bed to sleep at night for seven consecutive nights, concurrent with the use of an actigraphy monitor. The EMEP can be removed if the participant needed to mobilize during the night but to be re-worn on returning to bed to sleep. Participants randomized to elastic HB were instructed to wear it circumferentially at forehead level when they go to bed to sleep at night for seven consecutive nights with concurrent use of an actigraphy monitor. The sleep aids and actigraphy monitor were to be removed upon awakening.

On the visit after completion of the intervention week, participants were asked to provide a five-grade Likert scale response to “since using the sleep aid for the last one week, I have slept better” (strongly agree, agree, do not know, disagree or strongly disagree) and to rate using an 11-point visual numerical rating scale (VNRS) rated from zero to ten (higher score, greater satisfaction) and their satisfaction with the use of allocated sleep aid. Participants were asked to circle their responses on the Case Report Form. Pregnancy outcomes were obtained from hospital records after delivery. Participants’ data were transcribed onto the Case Report Form. Quantitative actigraphy-derived data was concealed from participants and care providers. A new set of sleep aids were provided to each participant as allocated and reused nightly by them during the period of study until no longer fit for purpose. EMEP and elastic HB were purchased from a thrift store at a cost of US$2.00-3.00 for each set.

Outcomes measures

Primary outcome metrics were mean night sleep duration change measured by actigraphy monitor over seven consecutive nights pre-intervention and during the intervention. Secondary outcomes include maternal satisfaction with sleep aid and self-reported sleep quality as well as a mode of delivery, labor induction and indication, delivery blood loss, labor analgesia, birthweight, umbilical cord arterial pH, Apgar scores, and neonatal admission and indication.

Sample size calculation

Our primary outcome was a mean change in night sleep duration. There was no previous similar study on the use of EMEP for improving sleep in pregnancy. Studies on intensive care unit patients show total sleep time increases of 40%-60% [11-13]. Assuming an improvement of night sleep duration of 1 hour (20% of five hours, the trial cut-off and below our center’s six-hour median sleep duration in late pregnancy) [9] and a standard deviation of 1.1 hours [5], applying alpha of 0.05 and power of 80%, 20 participants were required in each arm. Factoring in a 20% drop-out rate, 25 participants in each arm were needed for a powered study.

Statistical analysis

Data were entered into a statistical software package SPSS (Version 23, IBM, SPSS Statistics). The paired t-test was used to analyze paired continuous data within trial arms, and across trial arms, the t-test for means with normal data distribution, the Mann-Whitney U test for non-normally distributed data or ordinal data, and Chi-square test for categorical data. Two-sided P-values were reported and p < 0.05 was regarded as significant. The primary analysis was on an intention-to-treat basis.

## Results

**Figure 1:**
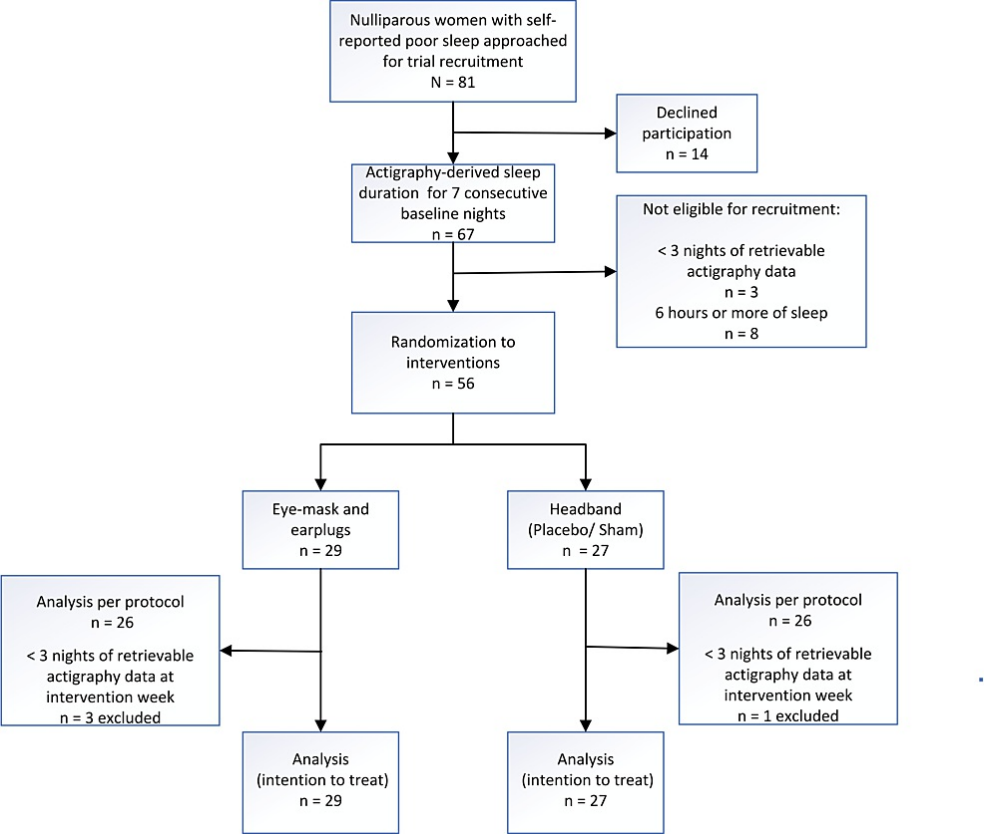
Recruitment flow chart of a randomized trial of eye masks and earplugs compared with headbands in nulliparas.

Figure 1 depicts the recruitment flow of participants throughout the study. Of the 81 potentially eligible women approached, 67 agreed to participate; 11 participants did not fulfill subsequent eligibility criteria (eight with mean night sleep ≥ six hours on actigraphy and three with less than three nights actigraphy data retrievable as per protocol requirement) [22], which left 56 to be randomized to EMEP (n = 29) and elastic HB (n = 27). Four participants in both EMEP (n = 3) and HB (n = 1) had less than three nights of retrievable actigraphy data during intervention week two (one in the HB arm had no retrievable data). We included all with actigraphy data availability for analysis based on the intention to treat principle although per protocol at least three nights of data out of seven consecutive nights [22] was required for the calculation of mean night sleep duration. We stopped recruitment on exceeding the targeted sample size.

Table 1 shows the characteristics of the trial participants including their bedroom environment which were similar across trial arms. The mean pre-intervention night sleep duration was 279 ± 19 minutes (4.7 ± 0.3 hours) vs. 286 ± 21 minutes (4.8 ± 0.4 hours) p = 0.17 and gestational age at recruitment was 35.3 ± 0.7 weeks vs. 35.0 ± 0.7 weeks p = 0.10 for EMEP and HB arms, respectively.

**Table 1:** Characteristics of nulliparous trial participants randomized to eye-mask and earplugs or headband (placebo) during night sleep. Data expressed as mean ± standard deviation or number (%). Analyses by Student t test for comparison of means for continuous data, Fisher’s exact test for 2x2 categorical datasets with any cell <5 and Chi-Square test for larger than 2x2 categorical datasets. Two-sided analyses P<0.05 for all variables. ^1^ No statistics computed as double bed is a constant.

Characteristics	Eye-mask-earplugs, n = 29	Headband, n = 27	P-value
Age (years)	30.6 ± 3.6	30.1 ± 3.3	0.56
Gestational age at recruitment (weeks)	35.3 ± 0.7	35.0 ± 0.7	0.10
Body mass index (kg/m2)	27.2 ± 3.1	27.9 ± 3.1	0.42
Retrievable night sleep data on Week 1	5.8 ± 1.2	5.6 ± 1.2	0.62
Ethnicity			
Malay	16 (55%)	17 (63%)	0.92
Chinese	9 (31%)	7 (26%)	
Indian	4 (14%)	3 (11%)	
Occupation			
Employment	21 (72%)	24 (89%)	0.33
Housewife and students	8 (28%)	3 (11%)	
House			
Bungalow	2 (7%)	1 (4%)	0.45
Semi-detached	7 (24%)	3 (11%)	
Terrace	9 (31%)	13 (48%)	
Condominium/ flat	11 (38%)	10 (37%)	
Room condition			
Fan	13 (45%)	10 (37%)	0.60
Air-conditioned	16 (55%)	17 (63%)	
Type of bed			
Double	29 (100%)	27 (100%)	.^1^
Single	0 (0%)	0 (0%)	
Night light	3 (10%)	3 (11%)	1.00

Table 2 reports actigraphy-derived night sleep duration metrics. For the primary outcome, the mean night sleep duration increased over baseline for the EMEP vs. HB were 25 ± 15 vs. 18 ± 17 minutes p = 0.13 was not statistically significant albeit greater prolongation with EMEP. Applying the paired t-test to evaluate the mean night sleep duration at baseline week compared to during intervention week, the mean increases of 25 ± 15 minutes (p = <0.001) in EMEP and 18 ± 17 minutes (p = <0.001) in HB arms were significant within both arms. Post hoc sensitivity analysis of the proportions of women who had their night sleep duration increased by at least 30 minutes were 13/29 (45%) vs. 5/26 (19%) RR 2.3 (95% CI 1.0-5.6) NNTb 4 95% CI 2.0-50.0, p = 0.04, being higher in EMEP.

**Table 2:** Primary outcome after randomization to eye-mask and earplugs or headband. Data expressed as mean ± standard deviation or number (%). Analyses by Student t test for comparison of means for continuous data, Fisher’s exact test for 2x2 categorical datasets with any cell <5 and Chi-Square test for larger than 2x2 categorical datasets. 2-sided analyses P<0.05 for all variables. ^1^ One participant in headband arm had no retrievable actigraphy Week 2 (intervention). ^2^ Paired test analysis comparing week 1 to week 2 within each trial arm.

Outcomes	Eye-mask-earplugs (n = 29)	Headband (n = 27)	RR (95% CI)	NNT_b_ (95% CI)	P-value
Mean night sleep duration (minutes)					
Week 1 (baseline)	279.0 ± 18.9	286.3 ± 20.9			0.17
		(n = 26^1^)			
Week 2 (intervention)	303.6 ± 18.8	301.9 ± 21.8			0.76
Week 1 & 2 increase (minutes)	24.7 ± 14.9	18.1 ± 17.3			0.13
	^2^p < 0.001	^2^p < 0.001			
Night sleep duration					
Any increase	28 (97%)	22 (85%)	1.1 (1.0-1.4)		0.12
≥ 30 minutes	13 (45%)	5 (19%)	2.3 (1.0-5.6)	NNT_b_ 4 (2.-50)	0.04
≥ 60 minutes	0 (0%)	1 (4%)			0.29

Table 3 shows the other secondary outcomes. Participants assigned to EMEP expressed higher satisfaction with their allocated sleep aid, median (interquartile range) score 7 (7.0-7.5) vs. 6 (5-7) p = 0.003 and were more likely to agree with the statement that they have slept better with their sleep aid 66% vs. 26% RR 2.2 (95% CI 1.1-4.6) p = 0.03, NNTb 3 95% CI 1.6-6.4 compared to participants assigned to HB. Labor induction rate was lower in the EMEP arm, 14% vs. 44% RR 0.3 (95% CI 0.1-0.8) NNTb 4 (95% CI 1.9-12.4), p = 0.02 with similar gestational age at delivery of 39.3 ± 1.0 weeks (EMEP) vs. 39.1 ± 1.2 weeks (HB) p = 0.44. There was no significant difference in the indications for labor induction between both arms, p = 0.17. Spontaneous vaginal delivery rates were 22/29 (77%) vs. 16/27 (58%), p = 0.25 and cesarean delivery rates were 5/29 (17%) vs. 7/27 (27%) p = 0.43 in EMEP and HB arms respectively. Other secondary outcomes were also not significantly different at the 5% level.

**Table 3:** Secondary outcomes after randomization to eye-mask and earplugs or headband. Data expressed as mean ± standard deviation, median (interquartile range) or number (%). Analyses by Student t test for continuous data, Fisher's exact test for 2x2 categorical datasets, Chi Square test for larger than 2x2 categorical datasets and Mann Whitney U test for non-parametric data (assessed by Kolmogorov-Smirnov test) or ordinal data. Two-sided P<0.05 for all variables. ^1 ^11-point visual numerical rating score (VNRS), with 0 representing completely dissatisfied and 10 representing completely satisfied. ^2 ^Recategorization of Likert scale responses: “agree” includes strongly or somewhat agree; “Do not agree” includes neither agree nor disagree, somewhat disagree and strongly disagree. ^3 ^Spontaneous vaginal delivery compared to operative delivery (instrumental vaginal and Cesarean delivery). ^4 ^Cesarean delivery compared to vaginal delivery (spontaneous vaginal and instrumental vaginal delivery).

Outcomes	Eye-mask-earplugs (n = 29)	Headband (n = 27)	RR (95% CI)	NNT_b_ (95% CI)	P-value
Nights of retrievable sleep data on Week 2	5.6 ± 1.8	5.6 ± 1.5			0.99
Participants’ satisfaction with sleep aid^1^	7 (7.0-7.5)	6 (5.0-7.0)			0.003
Likert Scale Questionnaire “Slept better with sleep aid”					
Agree^2^	19 (66%)	7 (26%)	2.2(1.1-4.6)	NNT_b_ 3 (1.6-6.4)	0.03
Do not agree^2^	10 (34%)	20 (74%)			
Maternal outcomes					
Gestational age at delivery (weeks)	39.3 ± 1.0	39.1 ± 1.2			0.44
Recruitment to delivery interval (weeks)	4.0 ± 1.2	4.1 ± 1.2			0.79
Mode of delivery					0.39
Spontaneous vaginal delivery	22 (77%)	16 (58%)	1.2(0.9-1.9)^3^		0.25^3^
Instrumental vaginal delivery	2 (7%)	4 (15%)			
Cesarean delivery	5 (17%)	7 (27%)	0.7(0.2-1.8)^4^		0.43^4^
Estimated blood loss during delivery (ml)	300 (225-350)	300 (300-400)			0.07
Induction of labor	4 (14%)	12 (44%)	0.3(0.1-0.8)	NNT_b_ 4 (1.9-12.4)	0.02
Indications of induction	(n = 4)	(n = 12)			
Gestational diabetes	2 (50%)	4 (36%)			0.17
Gestational hypertension		1 (9%)			
Prolonged latent phase		3 (27%)			
Premature rupture of membrane		2 (18%)			
Small for gestational age	1 (25%)	1 (9%)			
Post date	1 (25%)	1 (9%)			
Methods of induction					
Amniotomy	1 (25%)				0.01
Mechanical (Foley’s catheter)		8 (67%)			
Prostaglandins		4 (33%)			
Analgesia use in labor					
Epidural	8 (23%)	8 (30%)	0.8(0.3-1.8)		1.00
Pethidine	15 (52%)	12 (44%)	1.2(0.7-2.2)		0.61
Entonox	12 (42%)	13 (48%)	1.1(0.7-1.9)		0.79
Neonatal outcomes					
Birthweight (kg)	3.00 ± 0.30	3.14 ± 0.29			0.11
Apgar score at 1-minute	9 (9-9)	9 (9-9)			0.68
Apgar score at 5-minute	10 (10-10)	10 (10-10)			0.23
Umbilical cord arterial blood pH	7.32 ± 0.08	7.33 ± 0.09			0.73
	7.34 (7.25-7.35)	7.34 (7.26-7.40)			
Neonatal admission	3 (10%)	1 (4%)			0.61
Indications for neonatal admission	(n =3)	(n = 1)			
Infant of diabetic mother	1 (3%)				0.53
Transient tachypnea of newborn	2 (7%)	1 (4%)			

Post hoc, as we did not have a non-intervention arm to evaluate the Hawthorne effect of the sleep aids, we analyzed the mean night sleep duration on baseline week Day 1-3 compared to Day 5-7, 281 ± 26 minutes vs. 284 ± 28 minutes, p = 0.58 respectively and also Day 1 vs. Day 7 288 ± 27 minutes vs. 285 ± 29 minutes, p = 0.70, respectively, on a data availability basis (Tables 4, 5). There was no appreciable increase in baseline sleep duration later in the study to suggest a Hawthorne effect [23]. There was no major harm (e.g., major incident at home during night sleep) that occurred to participants during the trial.

**Table 4:** Post hoc Hawthorne-effect analyses for the entire trial population on baseline week data. Data expressed as mean ± standard. Analyses by paired t-test. ^1^ Cases used in analysis based on any data availability on the paired basis.

Outcomes	Prior to randomization	P-value
Week 1 mean night sleep (minutes)	n = 55^1^	
Day 1-3	281.1 ± 26.4	0.58
vs		
Day 5-7	283.6 ± 28.3	
	n = 29^1^	
Day 1	287.5 ± 27.1	0.70
vs		
Day 7	285.1 ± 28.6	

**Table 5:** Post hoc Hawthorne-effect analyses for trial population dichotomized to randomization to eye-mask and earplugs or headband intervention week data. Data expressed as mean ± standard. Analyses by paired t-test. ^1^ Cases used in analysis based on any data availability on the paired basis.

Outcomes	Eye-mask-earplugs	P-value	Headband	P-value
Week 2 mean night sleep (minutes)	n = 26^1^		n = 25^1^	
Day 8-10	308.6 ± 23.0	0.16	301.3 ± 32.3	0.83
vs				
Day 12-14	301.1 ± 25.8		299.6 ± 24.7	
	n = 17^1^		n = 10^1^	
Day 8	301.4 ± 32.3	0.60	309.3 ± 38.5	0.72
vs				
Day 14	307.4 ± 33.3		314.8 ± 20.2	

## Discussion

We find that both EMEP and HB increased actigraphy-derived mean night sleep duration compared to baseline in the late third trimester in nulliparous women with short sleep. Although the point estimate increase in mean night sleep duration of 25 vs. 18 minutes was higher with the EMEP arm, the difference was not significant. This finding that both EMEP and HB when labeled as sleep aids and used under such auspices lengthened sleep implied that if HB was a true sham, there was a significant placebo effect from it and the impact of EMEP was not significantly greater than sham/placebo impact of HB, highlighting the potential of suggestion in pregnant women. However, sensitivity analysis showed a significantly higher proportion of participants in the EMEP increased their night sleep duration by at least 30 minutes from baseline, agree that they slept better, and expressed higher satisfaction with allocated sleep aid when compared to the HB arm, thus suggesting an advantage for EMEP over sham HB on these metrics. Our positive findings with EMEP use in a home environment corroborated that of Hu et al. [14] who found the use of EMEP increased the duration of rapid eye movement sleep and subjective sleep quality in a simulated intensive care unit environment.

In our EMEP arm, the mean night sleep duration during the baseline week was 279 minutes with an increase of 25 minutes (9%). This increment is substantially less than the 40 to 60% [11,14] increment reported in the busy environment of the intensive care unit and the post-anesthesia unit [13], demonstrating the diminished but still efficacious sensory blockade of EMEP to improve sleep in the ambiance of the home bedroom.

Participants allocated to EMEP use expressed higher satisfaction compared to HB, in consistent with a previous study which had reported EMEP as a comfortable sleep aid and all participants used them easily [14]. Women in our EMEP arm compared to the HB arm were less likely to have their labor induced; visual inspection of the indications for induction showed prolonged latent phase, pre-labor rupture of membranes, and gestational diabetes as potential contributors to the difference. Better sleep has been shown to reduce labor duration [5], cesarean delivery [5], the incidence of gestational diabetes [6,7], and hypertension [7], so it is not implausible for EMEP to have these impact mechanisms towards reducing the labor induction rate. However, as our total sample size was only 56 women and our intervention was of only one-week duration, this finding was not powered and at risk of Type 1 error. We did not collect data on whether participants continued to use their allocated sleep aids after the trial to their delivery, a plausible occurrence as many women particularly in the EMEP arm subjectively reported they slept better and hence could be motivated to do that.

The EMEP vs. HB point estimates for spontaneous vaginal rates of 77% vs. 58% and cesarean delivery rates of 17% vs. 27% indicated the potential for EMEP to have a positive impact on these outcomes though these trends could have been driven by the labor induction rates differences as previously discussed above. In nulliparas undergoing induction of labor, short sleep in their last month of pregnancy is independently associated with a near doubling in cesarean delivery rate [9].

Sleep duration typically shortens and disordered sleep increases as pregnancy advances [4,24]. Hence, the night sleep duration in a later week should be shorter. However, we did not have a non-intervention control arm to evaluate this. If such a tendency was present, our sleep aids’ impact on sleep duration could be greater and there was no suggestion of a Hawthorne effect [23] in our data. We also did not collect data to evaluate whether HB was perceived to be a sham by participants.

We performed a PubMed (http://www.ncbi.nlm.nih.gov/pubmed) search on 01/19/2021 with the terms “earplug OR eye mask OR eye pad OR eye cover AND sleep trial” - 90 articles were retrieved. None of the articles relate to EMEP use at home or during pregnancy. Hypnotic drugs can have an adverse effect on the mother and fetus, and safety data on such drugs in pregnancy are limited [24,25]. A 2020 report on sleep pharmacotherapy for common sleep disorders in pregnancy showed that neonatal withdrawal is possible with drugs used in late pregnancy and non-pharmacologic interventions are preferred [26] implying an obvious role for an effective EMEP as a sleep aid.

Strengths and limitations

With regard to strengths, we performed an original proof-of-concept study on EMEP in a home bedroom environment, evaluated the placebo effect, and used the objective actigraphy monitor to quantify night sleep duration in pregnancy. Previous sleep studies in pregnancy often used subjective self-reported sleep [5-9,24]. A 2013 report on a comparison between self-reported and objective measures of sleep duration in pregnancy states that actigraphy is preferable to accurately assess sleep duration [27]. Our data collection was replete with only one participant completely without retrievable actigraphy data and our analysis was on the intention to treat.

As for limitations, the placebo effect with HB narrowed the sleep duration differences across the trial arms thus making our trial underpowered as we assumed a one-hour (20%) increase in night sleep for EMEP. We did not have data if participants used HB to cover their eyes or ears during night sleep. Our sample size was small and our intervention duration of one week was likely too short to provide meaningful power to evaluate clinically relevant outcomes of induction of labor, cesarean and spontaneous vaginal delivery. A powered study based on our pilot data on spontaneous vaginal delivery rates of 77% vs. 58%, would require a total sample of 166 for an EMEP vs. HB trial.

## Conclusions

The use of eye masks and earplugs in the home bedroom environment in nulliparous women with short night sleep in their late pregnancy resulted in a significant prolongation of their night sleep duration. Women also reported better night sleep quality and higher maternal satisfaction with eye masks and earplugs use as sleep aid. However, this study is not powered to evaluate other maternal outcomes. Therefore, further study is warranted to evaluate whether this low-cost, easy-to-use and well-tolerated sleep aid can improve pregnancy outcomes.
